# High frequency response of thick REBCO coated conductors in the framework of the FCC study

**DOI:** 10.1038/s41598-020-69004-z

**Published:** 2020-07-23

**Authors:** Artur Romanov, Patrick Krkotić, Guilherme Telles, Joan O’Callaghan, Montse Pont, Francis Perez, Xavier Granados, Sergio Calatroni, Teresa Puig, Joffre Gutierrez

**Affiliations:** 1 Institut de Ciència de Materials de Barcelona, C.S.I.C., Campus U.A. Barcelona, 08193 Bellaterra, Catalonia Spain; 2 ALBA Synchrotron—CELLS, Carrer de la Llum 2-26, 08290 Cerdanyola del Vallés, Barcelona Spain; 3 Universitat Politècnica de Catalunya, CommSensLab. c/ Jordi Girona 1, 08034 Barcelona Catalonia, Spain; 4 European Organization for Nuclear Research (CERN), 1211, Geneva 23, Switzerland

**Keywords:** Applied physics, Superconducting properties and materials, Materials science, Condensed-matter physics, Physics, Condensed-matter physics, Superconducting properties and materials

## Abstract

A thorough microwave response study of high temperature superconductors, considered as an alternative beam screen coating, has become integral in the design decisions for CERN’s future research infrastructure. Here, we present the surface resistance $R_{s}$ of various $\text {REBa}_{2}\text {Cu}_{3}\text {O}_{7-x}$ (RE = rare earth) coated conductors available in large scale as a function of magnetic field in a broad temperature range measured by a Hakki–Coleman type resonator with resonant frequency $\nu \approx {8}\,{\text {GHz}}$. Analysis of the high frequency dissipation supported by DC transport characterization reveals the vortex dynamics in thick $\text {REBa}_{2}\text {Cu}_{3}\text {O}_{7-x}$ films. Determined microscopic vortex parameters span over a wide range of magnitudes and reflect the relevance of the superconducting layer’s microstructure. We demonstrate that the depinning frequencies $\nu _0$ surpass $\nu$, which confirms the operation in high performing, low dissipation pinning regime at measurement conditions. Surface impedance extrapolation to FCC-hh conditions points towards a significant outperformance of copper by coated conductors in terms of surface resistance. The surface resistance margins would open up potential ways for a more efficient frontier circular collider.

## Introduction

Shortly after the discovery of high temperature superconductivity (HTS) in oxides by Bednorz and Mueller in 1986^1^, ceramic cuprates, in particular $\text {YBa}_{2}\text {Cu}_{3}\text {O}_{7-x}$ (YBCO), triggered universal euphoria among scientists and industry for the unprecedented high critical temperatures and sound performances under elevated magnetic fields. Decades of research activities later, second generation $\text {REBa}_{2}\text {Cu}_{3}\text {O}_{7-x}$ (REBCO, RE = Rare Earth), coated conductors (CCs) mark now the pinnacle in the industrialization of superconducting cuprates. The need for low grain boundary angles in these superconductors is accommodated by biaxially textured growth of $\sim \upmu \text {m}$ thick REBCO on functional oxide buffer layers backed by metallic tapes. It is a quite sophisticated and complex structure with practical use but expensive in production^2^. Most prominent applications take advantage of the well studied direct current (DC) or low frequency alternating current performance profile that identifies CCs as high current conductors at cryogenic temperatures and/or high fields^3–5^. The future circular hadron-hadron collider (FCC-hh), however, considers a novel utilization of superconductors reliant on the high frequency response of HTS at extremely challenging conditions^6^. The FCC-hh is the most ambitious scenario for a post Large Hadron Collider (LHC) machine proposed by CERN. It will operate as an $80 {-} 100 \,{\text {km}}$ acceleration ring where $16 {-} 18\,{\text {T}}$ magnets will steer $10^{11}$ protons in ${8}\, {\text {cm}}$ bunches with revolution period of ${0.3}\,{\text {ms}}$. In this machine, collision energies of $100 \,{\text {TeV}}$ at center of mass are expected to be reached. The $35.4\, {\text {W/m/beam}}$ synchrotron radiation emitted by the protons will be absorbed by a stainless steel tube, the so called beam screen, held at a temperature window of ${40 {-} 60}\,{\text {K}}$. The beam screen chamber is located in the beam-steering dipoles to thermally shield them. Given the prospected beam parameters, mirror charges will be induced into the beam screen that peak at magnitudes of ${25} \,{\text {A}}$ with frequencies up to ${1 {-} 2}\,{\text {GHz}}$. Governed by the necessity to maintain a stable beam during operation, highly conductive coatings will have to cover the interior of the beam screen chamber. Compared to copper, which has been the subject of a detailed study^7^, HTS promise with their lower surface resistance larger beam stability margins at expected operation temperature^8–10^. This motivates the need to understand and evaluate the high frequency response of HTS available on an industrial scale at FCC conditions. Our previous studies of six commercially available CCs have shown lower $R_s$ than ${300}\,\upmu {\text {m}}$ thick Cu colaminated on stainless steel (equivalent of FCC beam screen) at $T= {50}\,{\text {K}}$ and $\nu = {8} \,{\text {GHz}}$ up to ${9}\, {\text {T}}$. In addition, we demonstrated compatibility with thin a-C layers to mitigate the secondary electron yield^11^. In this work, we extend the temperature range of measured surface resistances to $T = 20 {-} 70 \,{\text {K}}$. It allows a discussion about the surface resistance and vortex physics of CCs with different microstructures in a wide range of temperatures. The vortex parameters depinning frequency $\nu _{0}$, vortex viscosity $\eta$ and Labusch parameter $k_{p}$ are derived within the Gittleman–Rosenblum and Bardeen–Stephen models. The model proposed by Gittleman and Rosenblum^12^ is a mean-field theory for vortices in a periodic pinning potential driven by high frequency oscillating, subcritical currents without thermal activation. It modulates both the resistive and reactive response of vortices to the driving field. In our case, the lack of complete surface reactance data sets has to be compensated by additional model confinements. With a modified Bardeen–Stephen model^13^, which describes the motion of vortices in a type II superconductor, we can estimate the vortex viscosity $\eta$. It reduces the Gittleman–Rosenblum model parameters to only one, the depinning frequency $\nu _{0}$, and thus makes it available through fitting the measured surface resistance $R_{s}$. We validate the two model approach for one sample with the ratio of surface reactance and surface resistance at $T = {20}\, {\text {K}}$, which lets us determine the depinning frequency sticking exclusively to the Gittleman–Rosenblum model. Finally, having established the microwave vortex parameters, we extrapolate the surface impedance of CCs down to $1 \,{\text {GHz}}$, up to $16\, {\text {T}}$ and compare it to the microwave response of Cu at FCC-hh conditions.

## Methods

Highly sensitive surface impedance measurements are performed with a high frequency, non-destructive, dielectric loaded resonator (DR) with resonant frequency $\nu \approx {8} \,{\text {GHz}}$. The resonator is based on the Hakki–Coleman geometry^14^, was previously used for non-linear characterization of HTS thin films^15,16^ and has been redesigned as described in^11,17^ in order to meet the sensitivity and geometrical requirements imposed by characterization of superconductors at cryogenic temperatures ($5 {-} 120 \,{\text {K}}$) and up to ${9} \,{\text {T}}$ in the ${25} \,{\text {mm}}$ bore of a *PPMS* from *Quantum Design*. The DR consists of a radiation shielding, cylindrical brass cavity terminated axially by interchangeable ${12\,{\text {mm}} \times 12 \,{\text {mm}}}$ samples and loaded with a single crystal rutile (TiO2) disk ($h = {3} \,{\text {mm}}$ and $\emptyset = {4} \,{\text {mm}}$), which is placed in the center of the cavity. The sample-dielectric-sample structure is sandwiched by copper-beryllium springs required to compensate contractions and expansions in the wide temperature range of measurements. The choice of dielectric material and cavity geometry results in a resonant frequency $\nu \approx {8}\,{\text {GHz}}$. Coupling loops through lateral walls are adjusted in such way that the resonator operates in the TE011 mode. This drives microwave currents to flow within the superconducting ${\text {CuO}}_{{2}}$ planes of the samples under investigation. Estimations of the RF field in the cavity yields $H_{\text {max}} = {28.68}\,{\text {A/m}}$ or ${0.036}\,{\text {mT}}$^18^. An RF power dependence study showed negligible (below 2%) increments in $R_s$ with respect to its zero-RF-field values at ${0}\,{\text {T}}$ external applied magnetic field (see supplementary notes). External magnetic fields are applied parallel to the c-axis of the superconductor. The unloaded quality factor $Q_{0}$ of the DR relates to the losses in the following way^17,19,20^:

1 $$\begin{aligned} \frac{1}{Q_{0}} = \frac{R_{\text {CC,1}}}{G_{\text {CC,1}}} + \frac{R_{\text {CC,2}}}{G_{\text {CC,2}}} + \frac{R_{\text {m}}}{G_{\text {m}}} + p \tan {\delta }. \end{aligned}$$

The first three terms of the sum are losses caused by the exposed conductive surfaces within the cavity, where the subscripts ‘CC,1’, ‘CC,2’ and ‘m’ stand for the coated conductors terminating the cavity and metallic enclosure, respectively. Here, *R* denotes surface resistances and *G* are the corresponding geometrical factors. The last term accounts for losses within the dielectric body. The dimensionless filling factor $p\approx 1$, since nearly the entire energy stored in the resonator is focused in the dielectric, and $\tan {\delta }$ is the loss tangent of rutile. In our geometry, losses of the metallic housing can be neglected^17^ and $\frac{R_{\text {CC,1}}}{G_{\text {CC,1}}} \approx \frac{R_{\text {CC,2}}}{G_{\text {CC,2}}} = \frac{R_{\text {CC}}}{G}$, thus

2 $$\begin{aligned} R_{\text {CC}}(T,B) = \frac{G}{2}\cdot \left[ \frac{1}{Q_{0}(T,B)} - \tan {\delta }(T)\right] . \end{aligned}$$

The procedures for numerical and empirical determination of *G* are described in^17^, whereas $\tan (\delta )(T)$ is adopted from^21^. In analogy to the surface resistance, the imaginary part of the surface impedance $Z_{\text {s}} = R_{\text {s}} + i X_{\text {s}}$ can be extracted from microwave measurements. The reactive response of a resonator reflects in the resonant frequency $\nu$ according to^20,22^:

3 $$\begin{aligned} -2\frac{\Delta \nu }{\nu _{\text {ref}}} = \frac{\Delta X_{\text {CC,1}}}{G_{\text {CC,1}}} + \frac{\Delta X_{\text {CC,2}}}{G_{\text {CC,2}}} + \frac{\Delta X_{\text {m}}}{G_{\text {m}}} + p \frac{\Delta \epsilon '_{r}}{\epsilon '_{r,\text {ref}}}, \end{aligned}$$

where *X* is the surface reactance, $\epsilon '_{r}$ the real part of the relative dielectric permittivity. By $\Delta x$ we mean the difference of a quantity from its reference value $x_{\text {ref}}$. Using the same approximations made already for the surface resistance, we get

4 $$\begin{aligned} \Delta X_{\text {CC}}(T,\Delta T,B,\Delta B) = -G \left[ \frac{\Delta \nu (\Delta T,\Delta B)}{\nu _{\text {ref}}(T,B)}+\frac{p}{2}\frac{\Delta \epsilon '_{r}(\Delta T)}{\epsilon _{r,\text {ref}}(T)}\right] . \end{aligned}$$

Magnetic field measurements at fixed temperatures are executed both in zero-field and field-cooled mode. Demagnetization of the superconducting external field magnet is followed after each field sweep to minimize the effect of trapped fields.

DC transport characterization is done either in van der Pauw geometry on $4 \,{\text {mm}} \times 4\, \text {mm}$ samples, or with well defined track bars of ${30}\,\upmu {\text {m}}$ width and ${200}\,\upmu {\text {m}}$ length. The width of the track bar ensures a cross section large enough to contain a representative number of grain boundaries. The tracks are defined by optical lithography. Temperature and magnetic field control are achieved, as described above, with a *Quantum Design PPMS*.

Subject of our study are seven commercially available CCs that differ in their microstructure. They are provided by *Bruker HTS GmbH*, *Fujikura Ltd.*, *SuNAM Co. Ltd.*, *SuperOx*, *SuperPower Inc.* and *THEVA Dünnschichttechnik GmbH*. An overview of all but one CCs’ microstructure, film thickness and growth method is given in^11^. In additional to the CCs in^11^, a new CC is provided by *Fujikura Ltd.* with nominal $I_c - \text {width} ({50}\, {\text {K}}) = {2}\,{\text {kA/cm}}- {\text {w}}$ and film thickness $t = {2.5}\, \upmu {\text {m}}$, where Eu is used as the rare-earth with ${\text {BaHfO}}_{{3}}$ (BHO) nano rods in the REBCO matrix. All RF characterization is done on CCs whose Ag and Cu protection layers are stripped off. In order to avoid degradation of the superconductor, the metallic layers are either chemically etched right before measurements or the CCs are provided stripped by the manufacturer and stored in a low humidity environment. The error on measured surface resistances arising from deviations in rutile centering and inhomogeneities in CC tapes is estimated to be ${10}\,{\%}$ as discussed in^11^, while the uncertainty in the surface reactance is, in a first approximation, assumed to result from the the error on resonant frequency $\sigma _\nu = \nu \cdot {1.5} \,{\text {ppm}}$. In the following, CCs are regarded without their protection layers and named according to their manufacturer.

## Results

### Surface resistance of thick CCs

In a general case, when exposed to a propagating microwave field, CCs’ entire complex architecture consisting of a thick superconducting layer with $t_{\text {SC}} \ge {0.9} \,\upmu {\text {m}}$ on top of a buffer layer stack with $t_{\text {dielectric}} > {0.5}\, \upmu {\text {m}}$ backed by a metallic, flexible substrate with $t_m \sim {50}\,\upmu {\text {m}}$ contributes to the microwave response. $t_{\text {layer}}$ stands here for the thickness of specified layers. Following transmission line analogy and impedance transformation^23^, the effective measured surface impedance of the CCs $Z_{\text {CC}}$ can be written as

5 $$\begin{aligned} Z_{\text {CC}}=Z_{\text {SC}} \frac{Z_{\text {stack}}+i Z_{\text {SC}}\tan (k_{\text {SC}} t_{\text {SC}})}{Z_{\text {SC}}+i Z_{\text {stack}}\tan (k_{\text {SC}} t_{\text {SC}})} , \end{aligned}$$

with

6 $$\begin{aligned} Z_{\text {SC}} = \sqrt{i2\pi \nu \mu _{0}{\tilde{\rho }}} \end{aligned}$$

being the surface impedance of the superconductor (valid in the local limit) and ${\tilde{\rho }}$ the complex resistivity^24,25^. $Z_{\text {stack}}$ is the effective measured surface impedance of the layers below the superconductor (dielectric buffer layers/metallic substrate) and $k_{\text {SC}} = \frac{\mu _{0}2\pi \nu }{Z_{\text {SC}}}$ the propagation constant of the superconductor. So, when interested in the characteristic surface impedance of the superconductor $Z_{\text {SC}}$, knowledge about the microwave response of the entire multilayer stack below the superconductor $Z_{\text {stack}}$ is necessary. However, Eq. (5) can be simplified if considering the characteristic screening lengths in the material under study. In the framework of the two fluid model, $|k_{\text {SC}} t_{\text {SC}}| \gg 1$ implies $Z_{\text {CC}} \approx Z_{\text {SC}}$, where the influence of the layers below the superconductor can be ignored^26^. This is known as the thick film approximation. $|k_{\text {SC}} t_{\text {SC}}|$ can be estimated for samples in our experiments. By assuming $T_{c} = {93}\, {\text {K}}$, $\rho _{n}({70}\,{\text {K}}) = {60}\,\upmu \Omega \,{\text {cm}}$ and $\lambda _{l}({0} \,{\text {K}}) = {150} \,{\text {nm}}$, typical values for YBCO, and $T = {70} \,{\text {K}}$, $\nu = {8} \,{\text {GHz}}$, as measurement conditions where the smallest propagation constant is expected, we get for our thinnest and thickest CC ($t_{\text {min}}\approx {0.9} \,\upmu {\text {m}}$, $t_{\text {max}}\approx {3.0} \,\upmu {\text {m}}$ ) $|k_{\text {min}} t_{\text {SC}}| \approx 5 > 1$ and $|k_{\text {max}} t_{\text {SC}}| \approx 16 > 1$, respectively, which does not clarify unambiguously if the thick film approximation is applicable. However, Silva et al.^26^ provide a numerical example for the deviation of the thick film approximation from Eq. (5). For ${1} \,\upmu {\text {m}}$ YBCO on a LAO single crystal substrate at $\nu = {30} \,{\text {GHz}}$ and $T = {72} \,{\text {K}}$, the error on the characteristic surface resistance by ignoring the substrate $R'_{\text {SC+LAO}} = R_{\text {SC}}$ is smaller than ${5}{\%}$. This error becomes even smaller when decreasing $\nu$. In conclusion, by applying the thick film approximation for the samples in this work, we make a systematic error on the characteristic surface resistance of the superconductor and all values derived from it that is smaller than 5%. With regard to specific microwave applications like the FCC-hh, however, the $Z_{\text {CC}}$ is still a very relevant quantity as it determines the effective microwave response of CCs in use.

We consider the expression for the experimental surface resistance as a function of RF field $H_{rf}$, externally applied flux density *B* and temperature *T*^8^:

7 $$\begin{aligned} R_{s}(H_{rf},B,T) = R_{BCS}(H_{rf},0,T) + R_{res}(H_{rf},0,0) + R_{fl}(H_{rf},B,T) \end{aligned}$$

with BCS formalism deduced component $R_{BCS}$ and the, in general, field and temperature independent residual surface resistance $R_{res}$ resulting from impurities and defects. $R_{BCS}$ usually considers only the temperature dependence^27^. The externally applied magnetic field dependence enters through $R_{fl}$ which represents the losses induced by the movement of fluxons when rendered to the mixed state^8,28^. Fluxons or vortices are understood as quantized magnetic field tubes with flux $\Phi _{0} = \frac{h}{2e}$ and a normal conducting core. For cuprates the vortex cores are of radius $\xi \sim \text {nm}$, known as the coherence length^29^. The last term is subject of many theories^12,30^ and will be later integral part of our analysis.

**Figure 1:**
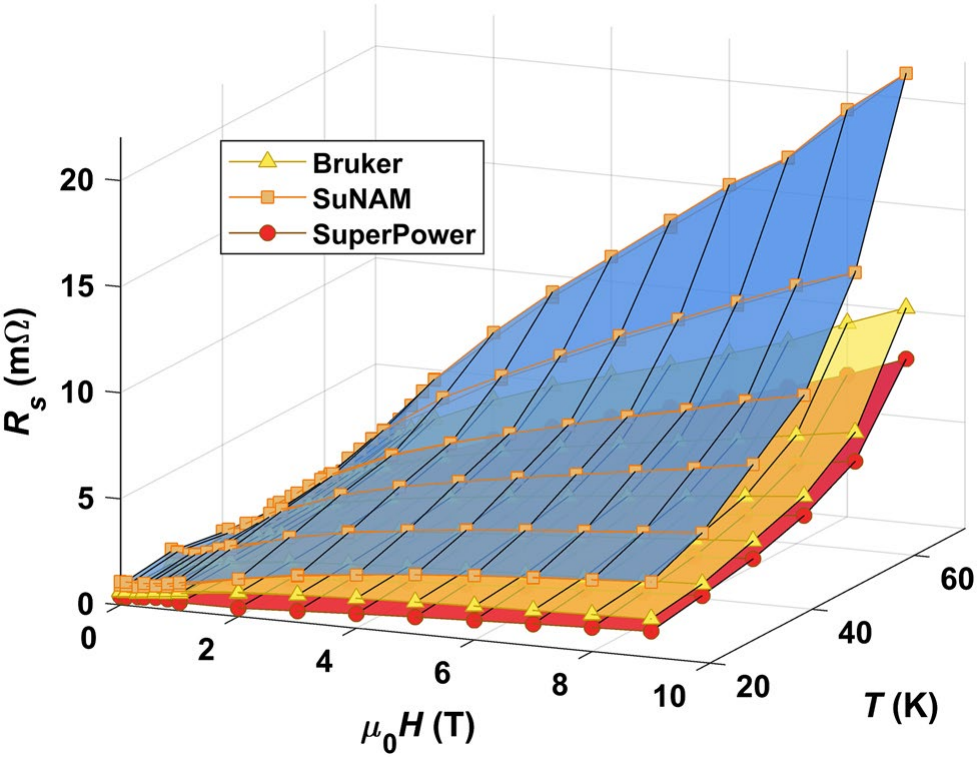
Absolute surface resistances $R_s$ of three selected coated conductors as a function of magnetic field at $T = 20 {-} 70\, {\text {K}}$. The markers (squares, triangles, circles) represent measurement points.

In Fig. 1 we show $R_{s}$ of three selected coated conductors as function of applied magnetic field $\mu _{0} H$ at several cryogenic temperatures *T*. SuNAM (as SuperOx and THEVA) represents a CC whose microstructure relies on an as grown pinning landscape with twin boundaries, stacking faults and point defects (pristine CC)^31^. Coated conductors with ’nanoengineered’ microstructures, meaning that secondary phases are deliberately incorporated into the REBCO matrix to enhance the pinning strength, are provided by Bruker, Superpower and Fujikura. Typically, nanoengineering can be realized in two ways. Either by adding artificial pinning centers (APC) made from nanosized oxides, e.g. $\text {BaZrO}_{3}$ (BZO) or BHO, in production processes or by establishing specific growth conditions that promote defects.

The corresponding CC microstructures control the field behavior of the surface resistance. The SuNAM sample has a steeper increase in $R_{s}$ with magnetic field $B\approx \mu _{0} H$ especially at mid and high *T* as compared to SuperPower or Bruker. The higher in-field performance of APC based superconductors known from critical current measurements^32,33^ is encountered here, too. This points towards an influence of APC on the surface resistance, hence, vortex dynamics where pinning is meaningful. Comparing Bruker with SuperPower, the field behavior at low and intermediate temperatures up to ${50} \,{\text {K}}$ is fairly similar. At higher *T* a slight difference in $R_{s}$ emerges, visible particularly at ${9}\, {\text {T}}$. It is in accordance with Bruker’s ’double disordered’ coined microstructure which is designed to pin vortices strongly at ${4.2} \,{\text {K}}$ and high fields at expense of performance at higher temperatures^34^. The features of the surface resistance’s field behavior that categorize the CCs into pristine and nanoengineered microstructure are observed for all measured samples. An overview of surface maps for all CCs can be found in the supporting information.

**Figure 2:**
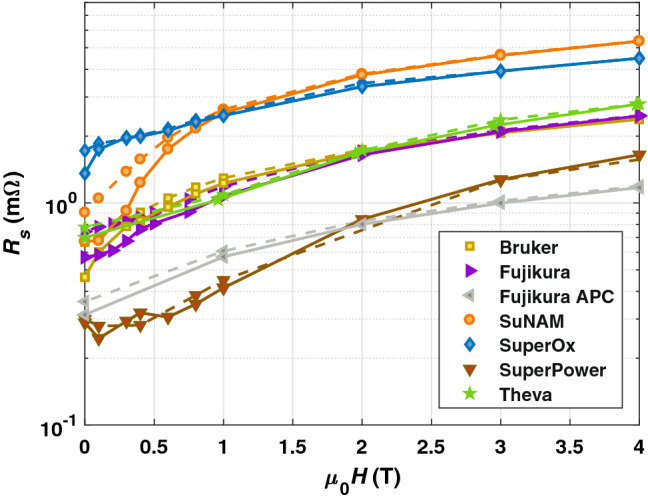
$R_s (H)$ of CCs measured at $T = {50}\, {\text {K}}$ and $\nu \approx {8} \,{\text {GHz}}$. The solid lines guide the zero-field cooled measurements, while the dashed lines connect the values recorded with field cooling. Below ${2}\,{\text {T}}$, a hysteretic behavior can be observed that points towards influences of trapped fluxons.

Figure 2 shows $R_{s}$ as a function of magnetic field at $T = {50} \,{\text {K}}$ for all considered CCs. A hysteretic behavior associated to field cooled/zero field cooled measurement procedures occurs at fields $< {2}\,{\text {T}}$, which indicates an influence of trapped vortices. In Table 1, we present $\frac{R_s^{\text {fc}} (B = {0}{\text {T}})-R_s^{\text {zfc}} (B = {0}{\text {T}})}{R_s^{\text {zfc}} (B = {0} {\text {T}})}$ for all measured samples. Interestingly, the hysteresis appears in different magnitudes for each CCs ranging between ${4}{\%}$ and ${44}{\%}$. A study about the effect of trapped field on the surface resistance of CCs and its relation to the superconducting layer’s architecture remains to be conducted. It might allow an optimization of CC technology targeting an improved field quality within the FCC-hh.

**Table 1:** Surface resistance hysteresis after exposing to ${9}\,{\text {T}}$ for all CCs at ${50}\,{\text {K}}$.

Provider	$\frac{R_s^{\text {fc}} (B = {0}{\text {T}})-R_s^{\text {zfc}} (B = {0}{\text {T}})}{R_s^{\text {zfc}} (B = {0}{\text {T}})}$
Bruker	44%
Fujikura	31%
Fujikura APC	15%
SuNAM	35%
SuperOx	27%
SuperPower	4%
Theva	10%

### Evaluation of microscopic vortex parameters

As described by Eq. (7) the microwave response with applied magnetic field is driven by the movement of fluxons in the superconductor. Consider a single vortex from an externally applied magnetic field $B = n \Phi _{0}$, *n* is the number of fluxons per unit area, in a type-II superconductor under a harmonic transport current density $\mathbf{J } = J_{0}\exp {(i2\pi \nu t)}\ \mathbf{n }_{a,b}$ with $J_{0}$ not close to the critical current density $J_{c}$. Here, $\nu$ is the current frequency in the range of $1 {-} 100 \,{\text {GHz}}$ and $\mathbf{n }_{a,b}$ a unit vector perpendicular to the applied magnetic field with unit vector $\mathbf{n }_{c}$. The vortex will be exposed to a Lorentz force $\mathbf{F }_{\text {L}} = \mathbf{J }\times \Phi _{0}\ \mathbf{n }_{c}$ exerted by the interplay with the electrical current. Under the presence of pinning sites, the vortex motion is constrained by pinning potential wells. This results in a pinning force $\mathbf{F }_{\text {p}} = -k_{p}\mathbf{x }$ which can be assumed to be linear to its vortex displacement $\mathbf{x }$. $k_{p}$ is the pinning constant or Labusch parameter encoding both the strength of pinning sites and vortex elasticity^25^. Additionally, the moving normal conducting vortex core gives rise to a viscous force $\mathbf{F }_{\text {v}} = -\eta \dot{\mathbf{x }}$, with $\eta$ being the vortex viscosity. It can be understood either as a conversion of Cooper pairs to quasiparticles where energy is lost^29^ or Joule dissipation within the vortex core^13^. By neglecting fluxon mass and thermal activation, the equation of motion for a single vortex is^24,25^

8 $$\begin{aligned} \eta \dot{\mathbf{x }}+k_{p}\mathbf{x } = \mathbf{J }\times \Phi _{0}\ \mathbf{n }_{c}. \end{aligned}$$

This equilibrium of forces is valid for not too large vortex displacements $\mathbf{x }$ in relation to the size of the potential wells within the superconductor. Moreover, nonlinearities are not considered in this model^24^. Gittleman and Rosenblum were the first to derive a response function to equation (8) in form of the complex vortex resistivity^12^

9 $$\begin{aligned} \rho _{\text {v}} =\rho _{\text {ff}} \frac{1}{1+i\nu _{0}/\nu }= \frac{B\Phi _{0}}{\eta }\frac{1}{1+i\nu _{0}/\nu }. \end{aligned}$$

$\nu _{0} = \frac{k_{p}}{2\pi \eta }$ denotes the depinning frequency, that is the material characteristic frequency above which the vortex response changes from the pinning to the flux-flow regime. The former is dominated by reactive, the latter by resistive contributions. At $\nu \ll \nu _{0}$ the vortex resistivity is completely imaginary with $\rho _{\text {v}} = -i2\pi \nu B \Phi _{0}/k_{p}$. $\nu \gg \nu _{0}$ reaches the extreme of the flux-flow regime in which $\rho _{\text {v}} = \rho _{\text {ff}}$. From Eqs. (6) and (9), we can express the surface impedance originating from fluxon movement as

10 $$\begin{aligned} Z_{fl} = Z(T,B)-Z(T,0) =\Delta Z= \sqrt{i2\pi \nu \mu _{0}\rho _{\text {v}}}. \end{aligned}$$

As shown in Figs. 1 and 2, it becomes evident that the vortex response, namely the field dependent part of the surface resistance dominates the total surface resistance under sufficiently high applied magnetic field. Hence, a good grasp of a superconductor’s in-field performance under the influence of microwaves can be gained by estimating the vortex properties $\eta$ and $\nu _{0}$, that are function parameters of Eq. (9).

We start with determining the vortex viscosity $\eta$. Bardeen and Stephen derived for the approximation of a superconductor in the ideal local limit ($l>\xi _{0}$) an expression for the flux flow resistivity $\rho _{\text {ff}}$. They assumed a normal conducting core of finite size with radius $\xi _{0}\sim \frac{1}{\sqrt{B_{c2}}}$. The flux flow resistivity is governed then by the normal state resistivity $\rho _{n}$ and the vortex core size, thus $\rho _{\text {ff}} \sim \rho _{n}\frac{B}{B_{c2}}$^13,29^. This approximation was derived for s-wave superconductors. With the time dependent Ginzburg–Landau theory, Ivlev and Kopnin modified the flux flow resistivity for YBCO by the factor $a = 1.45$ to account for the layered structure of the anisotropic uniaxial superconductor in the range $\mu _{0}H_{c1}\ll B \ll B_{c2}$^35,36^ :

11 $$\begin{aligned} \rho _{\text {ff}} =\rho _{n}\frac{B}{a\cdot B_{c2}}. \end{aligned}$$

Comparison of Eq. (11) with Eq. (9) yields for the vortex viscosity of REBCO superconductors

12 $$\begin{aligned} \eta = a\frac{\Phi _{0}B_{c2}}{\rho _{n}}. \end{aligned}$$

**Figure 3:**
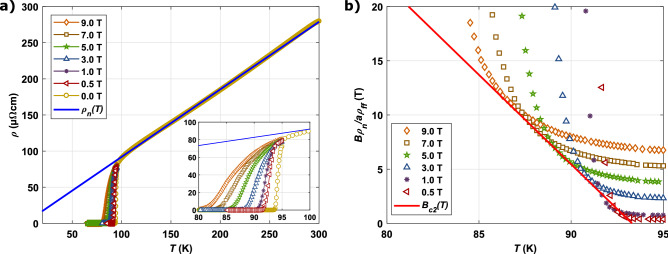
By way of example, data for Fujikura without APC is shown. (**a**) $\rho$ vs. *T* at various applied magnetic fields. The blue line is a linear fit to $\rho (T)$ well above $T_{c}$ which is used to estimate $\rho _{n}$. The inset zooms into the range where $\rho = \rho _{\text {ff}}$. (**b**) The ratio $B\rho _{n}/1.45\rho _{\text {ff}}$ as a function of temperature, as determined by flux flow scaling. Solid red line in the figure represents the upper critical field $B_{c2}(T)$.

A possible way to determine the vortex viscosity is by DC transport measurements from which the normal state resistivity $\rho _{n}$ and upper critical field $B_{c2}$ of the superconductors can be estimated. In Fig. 3a, we show the resistivity as a function of temperature in the range $T = {85 - 300}\, {\text {K}}$ at different applied magnetic fields up to ${9}\, {\text {T}}$ for one exemplary CC. The magnitude of $\rho$ follows values reported in literature^2^. $\rho _{n}$ below $T_{c}$ can be characterized in a first approximation from a linear extrapolation of the metallic behavior well above the critical temperature $T_{c}$. It was demonstrated in^37^ that the free flux-flow model applies for a considerable part of the resistive transition. For this reason, we can determine the magneto resistance measured close to $T_{c}$ as $\rho (T\approx T_{c},B) = \rho _{\text {ff}}(T,B)$. According to Eq. (11), the envelope of $\frac{B\rho _{n}(T)}{a\rho _{\text {ff}}(T)}$ represents points where $\rho (B) = \rho _{\text {ff}}(B)$ and gives an estimate for $B_{c2}(T)$ as shown in Fig. 3b for an example CC. As proposed in the theory of 3-D GL superconductors^29^, the upper critical field follows a linear temperature dependence $\beta (T_{c}-T)$, with $\beta = {1.6 {-} 1.9}\,{\text {T/K}}$ depending on characterized CC. The slope coincides with values $\beta = {1.9 {-} 2.1} \,{\text {T/K}}$ given in previous reports^37,38^. Even below $T= {78}\, {\text {K}}$, where the upper critical field of YBCO crosses over to 2-D behaviour with $B_{c2}\sim (T_c-T)^{1/2}$^29^, our estimates of $B_{c2}({20}\, {\text {K}})= {115 {-} 135} \,{\text {T}}$ from the linear extrapolation yield reasonable accordance with reported values $B^{\text {rep.}}_{c2}({20}\,{\text {K}})\approx {120}\,{\text {T}}$^39^.

**Figure 4:**
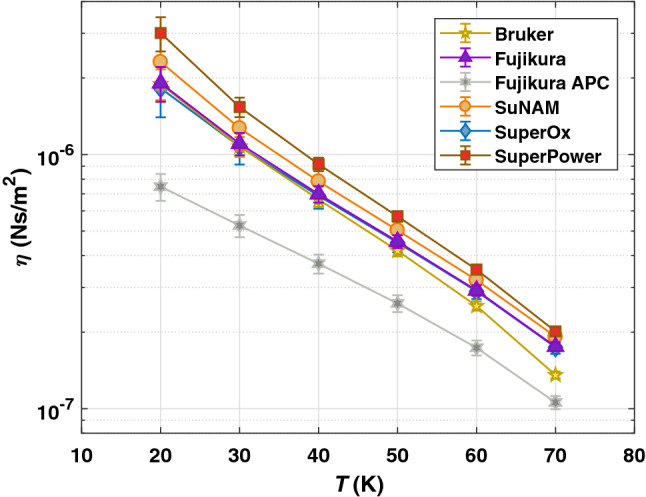
Vortex viscosity for all CCs but Theva determined from Eq. (12) at $T = {20 {-} 70}\, {\text {K}}$. The solid lines are guides for the eyes.

Eq. (12) is fed with estimated $\rho _{n}$ and $B_{c2}$ in order to calculate the vortex viscosity for measured temperatures. The values are presented in Fig. 4 for all CCs but Theva. Vortex parameters of samples from THEVA Dünnschichttechnik GmbH are not presented in this article. The inclined REBCO c-axis in Theva CCs requires the consideration of anisotropy in quasiparticle and/or vortex dynamics which goes beyond the scope of presented elaborations and deserves a separate study. Error bars are derived from the determination of $\rho _{n}$ and $B_{c2}$ and then propagated to $\eta$. The vortex viscosity is for all samples inversely proportional to the temperature. While the magnitudes of $\eta$ for most of the CCs are within the errors relatively close together, the sample Fujikura with APC significantly undercuts the other CCs by a factor of up to 4 depending on the temperature. Since $\eta$ relates to the conversion of cooper-pairs to quasiparticles and vice versa of a moving flux line^40^, the entire microstructure of a CC may contribute to $\eta$ and explain the variety between values of different providers.

**Figure 5:**
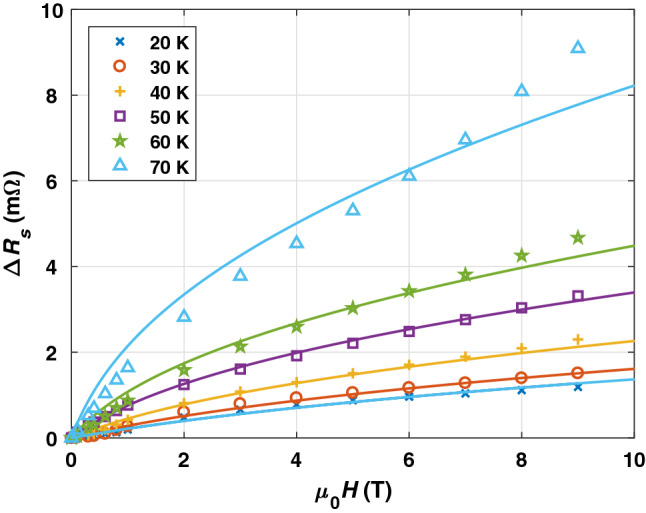
The markers show the dissipation under microwaves of $\nu \approx {8}\,{\text {GHz}}$ with applied magnetic field $\mu _{0} H$ up to ${9}\, {\text {T}}$ at different temperatures of a Bruker CC. Corresponding fits with real part of equation (10) are represented by solid lines. The lines follow the same color code as the markers.

The second vortex parameter $\nu _{0}$ in applied magnetic field can now be extracted from Eq. (10). When constraining the Gittleman–Rosenblum model with the determined vortex viscosities (see Fig. 4), we can employ $\nu _{0}$ as a fitting parameter to the measured field response of the surface resistance $R_{fl}(B) = \Delta R_{s}$. By way of example, $\Delta R_{s}$ of a Bruker sample in the range $T = {20 {-} 70}\,{\text {K}}$ together with the real part of Eq. (10) fitted to each data set of a corresponding temperature is shown in Fig. 5. Up to ${50} \,{\text {K}}$, $\Delta R$ increases quite steadily with $\sqrt{B}$ as described by the Gittleman–Rosenblum model. At higher temperatures, especially at high fields, measurement data deviates from the predictions with a steeper increase than $\sqrt{B}$. The Gittleman–Rosenblum model is limited to the mean-field regime, that is where the displacement of vortices from the pinning potential minima is small enough so that the flux tubes stay inside the wells and vortex–vortex interaction is infinite, resulting in a collective movement as a lattice^24,25^. There are two possible explanations for the observed model deviation. On the one hand, thermal activation can excite hops between pinning sites, which a mean-field model cannot account for^41^. On the other hand, at high fields we are approaching $B_{irr}$. For example, $B_{irr}(T= {70}\,{\text {K}})\approx {16}\,{\text {T}}$ for Bruker. In this case, the long-range order gets disturbed and the infinite vortex–vortex repulsion of mean-field models loses its validity.

**Figure 6:**
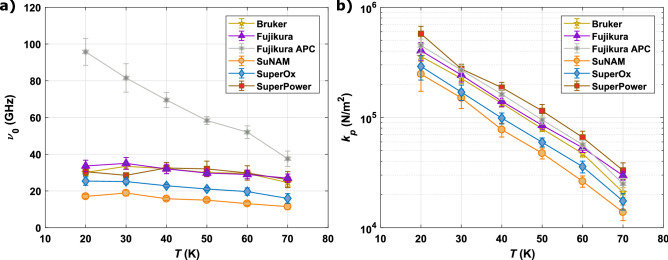
(**a**) Depinning frequency determined from fits with real part of Eq. (10) and (**b**) Labusch-parameter for all CCs determined from the relation $k_{p} = 2\pi \nu _{0}\cdot \eta$ at $T = {20 {-} 70}\, {\text {K}}$ in ${10} \,{\text {K}}$ steps. Error bars for $\nu _{0}$ are derived from the quality of the fittings and are propagated together with uncertainty of $\eta$ to the error of $k_p$. The solid lines are guides for the eye.

Figure 6a shows the depinning frequency $\nu _{0}$ extracted from fits with Gittleman–Rosenblum model for all CCs at $T = {20 - 70} {\text {K}}$. Except for Fujikura with APC, the deviation of depinning frequencies as function of temperature is quite small and inversely proportional to temperature. A correlation between microstructure and depinning frequency can be observed. Bruker (double disorded), SuperPower (BZO nanorods) and Fujikura APC (BHO nanorods) characterized by the use of artificial pinning centers exhibit increased depinning frequencies as compared to SuNAM or SuperOx CCs that rely on as grown pinning. Remarkably, Fujikura without APC has within the errors the same depinning frequency as Bruker and SuperPower at all temperatures which points towards a nanoengineered microstructure that resembles the pinning properties of both CCs with APC. The determined values for all but one CC remain in accordance with literature which report mostly $\nu _{0} = {10 {-} 50} \,{\text {GHz}}$ in the given temperature range (see^25,42^ and references within). The depinning frequency of Fujikura with APC exceeds conventionally measured $\nu _{0}$ both in magnitude and steepness of the temperature dependence. As already mentioned above, Fujikura with APC is the only CC that incorporates Eu as the rare earth and BHO as the nano additive. Whether these two changes are the governing factors for the increased depinning frequencies cannot be concluded unambiguously without additional studies.

**Figure 7:**
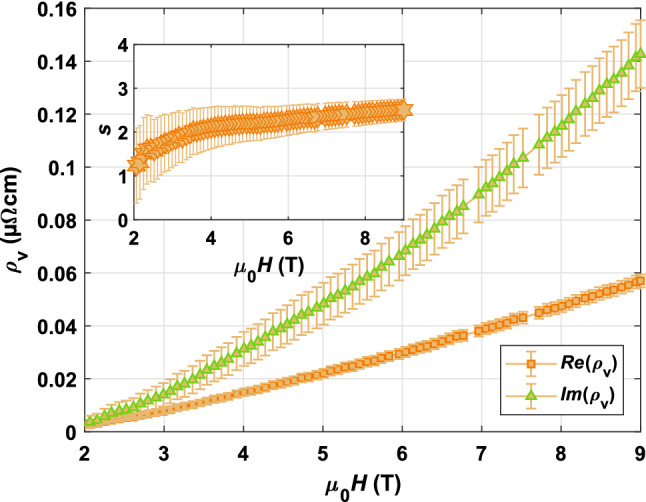
The complex vortex resistivity of the bottom surface of SuNAM as a function of applied magnetic field at $T = {20}\,{\text {K}}$. The inset shows the corresponding ratio $s = \frac{\text {Im}(\rho _{\text {v}})}{\text {Re}(\rho _{\text {v}})} = \frac{\nu _{0}}{\nu }$ as a function of magnetic field. The errors on $\rho _{\text {v}}$ and *s* are propagated from errors on the surface resistance $\Delta R_s$ and resonant frequency $\nu$.

In order to determine the depinning frequency $\nu _{0}$ within the Gittleman–Rosenblum model only, the resistive and reactive part of the surface impedance are needed. It gives rise to the ratio $s = \frac{\text {Im}(\rho _{\text {v}})}{\text {Re}(\rho _{\text {v}})} = \frac{\nu _{0}}{\nu }$ and thus the determination of depinning frequency $\nu _{0}$ independent of the vortex viscosity $\eta$. We measured the increase of surface reactance with magnetic field $\Delta X_s = X_s(T,H)-X_s(T,0)$ for one CC at $T = {20} \,{\text {K}}$. As already discussed and seen from Eq. (3), contributions to the reactive response of a resonator can also arise from the dielectric. During the measurement of $\Delta X_s$, we made carefully sure to eliminate those contributions by reaching a temperature stability $\Delta T$ with which $\Delta \epsilon '_{r}(\Delta T) \approx 0$ (see Eq. (4)). Figure 7 presents $\rho _{\text {v}}$ for a SuNAM CC at $T = {20} \,{\text {K}}$ and high fields. The real part of the vortex resistivity follows, as expected, a linear field behavior. The upward curvature in $\text {Im}(\rho _{\text {v}})$ suggests a field behavior in the vortex parameters^43^. From the inset of Fig. 7, the depinning frequency can be estimated as a function of magnetic field and is in the range $\nu ^{\text {SuNAM}}_{0}({20}\,{\text {K}}, {4 {-} 9} \,{\text {T}}) \approx {16.3 {-} 19.3} \,{\text {GHz}}$. The excellent agreement with $\nu ^{\text {SuNAM}}_{0}({20}\,{\text {K}}) \approx {(17.31 \pm 1.25)}\, {\text {GHz}}$ determined from a Gittleman–Rosenblum model fit with constrained $\eta$, as presented in Fig. 6a, reinforces the legitimacy of our approach for vortex viscosity estimations.

Both, the obtained values for the depinning frequency as presented in Fig. 6a and the ratio $s = \frac{\text {Im}(\rho _{\text {v}})}{\text {Re}(\rho _{\text {v}})} > 1$ point towards pinning based vortex dynamics of the CCs at measurement conditions. For all characterized CCs at least up to $T = {60} \,{\text {K}}$ and ${9}\,{\text {T}}$, the measurement frequency $\nu$ is well below the depinning frequency $\nu _{0}$. Thus, we would expect the reactive part of the surface impedance to be dominant over the resistive part, which is, in fact, observed for SuNAM at $T = {20}\,{\text {K}}$ in Fig. 7. These conditions indicate operation in the so called pinning regime where the microwave response is low in dissipation and the surface resistance is governed by the Labusch parameter $k_{p}$, consequently by the pinning forces. Phenomenologically speaking, at low frequencies vortices are displaced from their potential minimum far enough to ’see’ the pinning well. It contrasts with the flux-flow regime. In the flux-flow regime, where $\nu >\nu _{0}$, oscillations of flux tubes are so small that the pinning potential becomes invisible to the nanometric fluxon movements. The fluxons dissipate in their potential minimum as if no pinning potential was present. In this case, the vortex viscosity $\eta$ mainly contributes to $\Delta R$^25^.

The Labusch parameter $k_{p} = 2\pi \nu _{0}\cdot \eta$ is presented as a function of temperature for all CCs in Fig. 6b. Here, the CCs’ different microstructures are reflected again quite well. Bruker, SuperPower and both Fujikura samples, defined as CCs with high pinning microstructure, exhibit over the entire *T* range increased $k_{p}$ as compared to as grown pinning CCs. As described above, the magnitude of measured surface resistances $R_{s}$ are mostly governed by the Labusch parameter $k_{p}$ at measurement conditions, meaning CCs with small $k_{p}$ display high surface resistances.

Uncertainties in the determination of pinning parameters given in the figures originate from estimated errors on $R_s$, $\nu$ and goodness of fits. While they are significant in the comparison of the CCs, they do not account for the uncertainties of the intrinsic vortex parameters introduced by the pure choice of a model. In all the analyses, we have neglected flux creep effects which may be relevant especially at elevated temperatures. It has been reported that by neglecting the flux creep, the real part of the intrinsic microwave response is overestimated. Thus, the depinning frequency and Labusch parameter derived here within the Gittleman–Rosenblum model may represent lower limits^43^.

### Extrapolation of surface impedance to FCC conditions

Transverse beam coupling between accelerated hadrons and the introduced image currents within the beam screen coating pose one of the performance limitations in the current FCC-hh design. The transverse beam coupling impedance is directly proportional to the surface impedance of the material facing the particle beam^9,44^. In a previous work, we compared the surface resistance of the potential FCC-hh beam screen coating Cu with the CCs presented here. At $T = {50}\, {\text {K}}$, $\mu _{0}H = {9}\,{\text {T}}$ and $\nu \approx {8}\,{\text {GHz}}$, the best performing CC has a 2.5 smaller surface resistance than Cu^11^. Aforementioned work and previous discussions enable extrapolation of the surface impedance of Cu and CCs to FCC-hh conditions. The microwave response of ${300} \,\upmu {\text {m}}$ thick Cu colaminated on stainless steel by CERN is measured with the presented dielectric resonator and amounts $R^{\text {FCC Cu}}_{s}({50}\,{\text {K}}, {0}\,{\text {T}}, {8}\,{\text {GHz}}) \approx {7.5}\,{\text {m}}\Omega$^11^. Since the surface impedance of a normal, non-magnetic metal is related to its skin depth^44,45^

13 $$\begin{aligned} \delta = \sqrt{\frac{2\rho }{2\pi \nu \mu _{0}}} \end{aligned}$$

by

14 $$\begin{aligned} Z^{\text {metal}}_{s} = (1+i) \frac{\rho }{\delta }, \end{aligned}$$

we can deduce the surface resistance of Cu at FCC conditions to be $R^{\text {FCC Cu}}_{s}({50}\,{\text {K}}, {0}\,{\text {T}}, {1}\,{\text {GHz}}) = \sqrt{\frac{{1}\,{\text {GHz}}}{{8}\,{\text {GHz}}}} \cdot R^{\text {FCC Cu}}_{s}({50}\,{\text {K}}, {0}\,{\text {T}}, {8}\,{\text {GHz}}) \approx {2.65}\,{\text {m}}\Omega$. The change with applied magnetic field is insignificant. As indicated by Eq. (14), $R_{s} = X_{s}$ for normal conductors.

**Figure 8:**
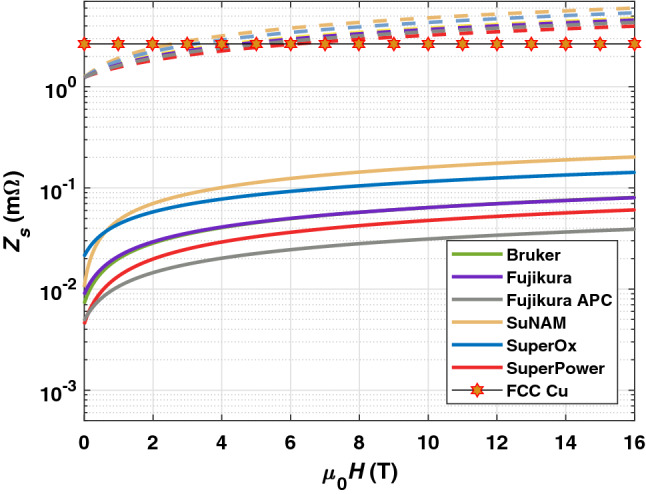
To $T = {50}\,{\text {K}}$ and $\nu = {1}\,{\text {GHz}}$ extrapolated surface impedance for ${300}\,\upmu {\text {m}}$ thick Cu sputtered on stainless steel, denoted here as FCC Cu, and several CCs as a function of magnetic field. The stars represent both the surface resistance and surface reactance values of FCC Cu. The solid lines are surface resistances, the dashed lines surface reactances of CCs. The provider of the CCs are color coded.

Returning to Eq. (7), the field independent part for the surface resistance $R_{\text {BCS}}$ of a superconductor in the dirty limit (short mean free path) has a quadratic frequency dependence for $T<T_{c}$ and not too high frequencies ($2\pi \nu \ll 2\Delta /\hslash$, $\Delta$ is the energy gap)^46^. $R_{\text {res}}$ can exhibit different frequency dependencies^47^, however, in high $T_c$ superconductors quadratic frequency behaviors were observed^46,48^. In this setting, it can be considered $R^{\text {CC}}_{BCS}({50}\,{\text {K}}, {0}\,{\text {T}}, {1}\,{\text {GHz}})+R^{\text {CC}}_{res}({50}\,{\text {K}}, {0}\,{\text {T}}, {1}\,{\text {GHz}}) = \left( \frac{{1}{\,\text {GHz}}}{{8}\,{\text {GHz}}}\right) ^2 \cdot R^{\text {CC}}_{s}({50}\,{\text {K}}, {0}\,{\text {T}}, {8}\,{\text {GHz}}) \approx {10}\,{\upmu \Omega }$. The field independent part of the surface reactance can be written within the BCS theory as $X_{\text {BCS}} = 2\pi \nu \mu _{0}\lambda _{l}$, where $\lambda _{l}$ stands for the London penetration depth. Assuming the conventional temperature dependence^29^

15 $$\begin{aligned} \lambda _{l}^{2} = \frac{\lambda _{l}^{2}({0}{\text {K}})}{1-\left( T/T_{c}\right) ^{4}} \end{aligned}$$

with $\lambda _{l}({0}{\text {K}}) = {150}\,{\text {nm}}$^2^ and $T_{c}$ the critical temperature measured by transport measurement of corresponding CCs, we can estimate the expected surface reactance $X_{BCS}(T = {50}\,{\text {K}}, \nu = {1}{\text {GHz}})\approx {1.2}\,{\text {m}}\Omega$. Finally, the field dependent part of the superconductor’s surface impedance at FCC conditions is calculated from Eq. (10) and estimated pinning parameters at $T = {50}\,{\text {K}}$. A discussion on the limits of this approach is given in the supplementary notes. In Fig. 8, we present surface impedance extrapolated to FCC conditions as a function of magnetic field for FCC Cu in comparison with all CCs covered in this work. The surface resistance of the CCs at ${16}\,{\text {T}}$ is expected to be smaller than that of Cu by a factor of 15–70 depending on the provider. The surface reactance of CCs exceeds that of Cu by a factor of 1.5–2.3. So, the use of current state of the art CCs in the beam screen chamber results in highly decreased surface resistance with the trade off of slightly increased reactive microwave response as compared to Cu. Realized in practice, it would mean significant benefits in terms of beam stability margins, resistive power losses and possibly a minor maximum beam current limitation within the FCC^49^. Furthermore, the CC technology offers room for optimization. As pointed out in the previous section, CCs under study perform at FCC conditions in the pinning regime. Hence, from an engineering point of view, it is desirable to use superconductors with large pinning constants $k_{p} = 2\pi \nu _{0}\cdot \eta$, that is, large depinning frequency and vortex viscosity for this particular application. As shown in Fig. 6a, incorporation of artificial pinning centers have positive impacts on the depinning frequency. The vortex viscosity $\eta$ can be increased by either decreasing the normal state resistivity $\rho _{n}$ or increasing the upper critical field $B_{c2}$ as indicated by Eq. (12).

## Conclusions

We have studied the surface resistance, $R_s$, and vortex properties ($\nu _0$, $\eta$ and $k_p$) at microwave frequencies of seven commercially available REBCO coated conductors as a function of magnetic field up to ${9}\,{\text {T}}$ at $\nu = {8}\,{\text {GHz}}$ and ${20 {-} 70} {\text {K}}$. We have revealed that these magnitudes correlate strongly with the microstructure of the superconducting layer, elucidated the vortex dynamics at high frequencies and confirmed the high microwave performance of CCs for large scale applications.

We have calculated the surface impedance of CCs at FCC conditions ($T = {50}\,{\text {K}}, \nu = {1}\,{\text {GHz}}$ and ${16}\,{\text {T}}$) by means of the Gittleman–Rosenblum model. Results suggest that as compared to Cu colaminated on stainless steel, the surface resistance is expected to be a factor 15–70 smaller (depending on the provider), while the surface reactance stays in the same order of magnitude.

The estimated outstanding microwave performance of CCs at FCC conditions encourage to take next steps in the feasibility study. Namely, to determine experimentally the surface impedances at ${1}\, {\text {GHz}}$ and ${16}\, {\text {T}}$, study the different ways to attach tens of meter-long CCs to the beam screen chamber taking into account the mechanical properties of the stainless-steel/CC ensemble, ensure magnetic field quality inside the beam-screen chamber, and study possible hazards such as a dipole magnet quench. These factors will be examined in detail as they represent essential puzzle pieces for a final, reliable cost-benefit assessment for this solution.

In summary, our manuscript highlights explicit relations between superconducting properties and microscopic vortex parameters to tailor CCs for specific microwave applications, and promotes further considerations for employing CCs as a beam screen coating alternative to copper for the FCC-hh.

## Electronic supplementary material

Supplementary informaiton.

## Data Availability

Raw data was generated at ICMAB-CSIC. Raw and derived data supporting the findings of this study are available from the corresponding author T.P. on request.
