# Supplementary material for: High frequency response of thick REBCO coated conductors in the framework of the FCC study
Source: Sci Rep. 2020 Jul 23;10:12325. doi: 10.1038/s41598-020-69004-z (PMC7378233; doi:10.1038/s41598-020-69004-z)
Supplement: Supplementary file 1 — Supplementary informaiton. [file 41598_2020_69004_MOESM1_ESM.pdf]

High frequency response of thick REBCO Coated Conductors in the framework of the FCC study

Artur Romanov[1,*], Patrick Krkotić[2,3], Guilherme Telles[1], Joan O'Callaghan[3], Montse Pont[2], Francis Perez[2], Xavier Granados[1], Sergio Calatroni[4], Teresa Puig[1,], and Joffre Gutierrez[1]**

[1]Institut de Ciència de Materials de Barcelona, C.S.I.C., Campus U.A. Barcelona, E-08193 Bellaterra, Catalonia, Spain
[2]ALBA Synchrotron—CELLS, Carrer de la Llum 2-26, E-08290 Cerdanyola del Vallés (Barcelona), Spain
[3]Universitat Politècnica de Catalunya. CommSensLab. c/ Jordi Girona 1, E-08034 Barcelona, Catalonia, Spain
[4]European Organization for Nuclear Research (CERN), 1211 Geneva 23, Switzerland
[*]aromanov@icmab.es
[**]teresa.puig@icmab.es

Supplementary Notes

Supplementary Note 1: Surface resistance maps of all CCs

The categorization into pristine (SuperOx, SuNAM) and nanoengineered CCs (Bruker, Fujikura, SuperPower, Theva) described in the main text are reinforced by Fig. S1. Dedicated comments require the samples of SuperOx and Theva. SuperOx suffered an constant increase of $R_s^{\text{offset}} \approx 5\,\text{m}\Omega$ as compared to the values published in[1]. We subtracted this offset, since we can attribute it to an insufficient removal of the Ag protection layer which influenced the measurement. Remarkably, Theva performs in terms of $R_s(\mu_0 H)$ quite similar to samples with APC without incorporating them. One reason for the increased in-field performance could be Theva's inclined substrate technology which induces a tilt of REBCO's c-axis by 24° to the tape normal during the growth process[2]. For a given applied magnetic field pointing perpendicular to the substrate plane, the effective field along the c-direction, hence the Lorentz force within the ab-planes, is reduced as compared to other CCs with c-axes normal to the tape. In addition, we compare colaminated Cu on stainless steel as prospected to be used in the FCC beam screen (denoted as FCC Cu) with all CCs in terms of R_s in Fig. S1. FCC Cu was measured at zero field and assumed to have no magnetoresistance. At 8 GHz, 50 K and 9 T, nanoengineered CCs exhibit $R_s^{\text{nano.}} \approx 0.25 - 0.5 \cdot R_s^{\text{FCC Cu}}(50\,\text{K})$. For pristine CCs at 8 GHz, 50 K and 9 T, $R_s^{\text{pristine}}(50\,\text{K}) \approx R_s^{\text{FCC Cu}}(50\,\text{K})$, while they have a lower surface resistance than Cu at smaller fields and lower temperatures. At FCC conditions (1 GHz, 50 K, 16 T) the performance advantage of CCs over Cu becomes more pronounced: depending on the provider it is expected to be by a factor of 15-70 smaller than the surface resistance of Cu.

Supplementary Note 2: Extrapolation of surface impedance to FCC conditions

Presented calculations for the field dependence of CCs' surface impedance are performed within the mean-field approximation which are very successful in elevated frequency ranges $\nu \geq 8\,\text{GHz}$[3]. Going to lower frequencies increases the excursion of the vortices, thus vortex-vortex interaction and thermal fluctuations become more relevant[4]. Close to conditions where the vortex-liquid transition is passed, data is better described by scaling models. Wu et al. found that the crossover frequency $\nu_x \sim 8 - 9\,\text{GHz}$ at $T = 80.2\,\text{K}$ and $H = 0.4\,\text{T}$ for YBCO[4]. It defines the frequency at which the liquid-glass transition occurs. The crossover frequency ν_x at $T = 50\,\text{K}$ remains unknown but is expected to be dependent on microstructure and lower than at 80.2 K since oscillations are less influenced by vortex-solid breaking flux creep.

Supplementary Note 3: Coated conductor architecture

Part of this study are seven coated conductors from six different providers. Table S1 shows characteristics of the corresponding REBCO layer that are significant to our analysis.

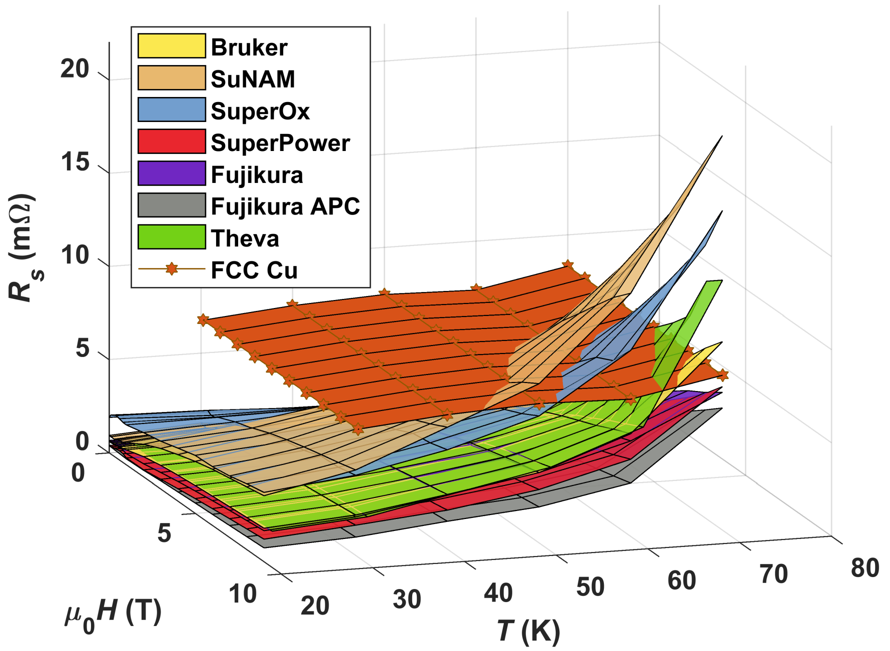

Figure S1. Absolute surface resistances R_s of all measured coated conductors as a function of magnetic field at $T = 20-70$ K. The crossings of lines represent measurement points.

Provider	Rare-earth	Nano-inclusions	Thickness (μm)
Bruker	Y	$BaZrO_3$	1.6
Fujikura	Gd	-	1.8
Fujikura APC	Eu	$BaHfO_3$	2.5
SuNAM	Gd	-	1.6
SuperOx	Gd	-	0.9
SuperPower	Gd, Y	$BaZrO_3$	1.5
Theva	Gd	-	3.0

Table S1. The thicknesses of the REBCO layers were determined with a profilometer and have been confirmed together with disclosed information by the providers.

Supplementary Note 4: Estimation of RF-field in cavity

Our estimations of the RF field in the cavity used in this work yielded $H_{max} = 28.68\,\mathrm{A/m}$ or $0.036\,\mathrm{mT}$[5]. For the FCC-hh, assuming a peak beam-induced image current of 25 A and a beam screen with diameter of 30 mm[6], the amplitude of the RF field at the surface of the beam screen has been estimated to be 250 A/m or 0.3 mT, a factor 10 larger than the value obtained in the measurements presented. We have performed a RF field power dependence study of the surface resistance up to powers of 1000 A/m at zero applied field for one provider and it shows a 2% increase in surface resistance at FCC-hh field amplitude with respect to small RF-field values, see Fig. S2. For the samples measured, the surface resistance at FCC-hh field amplitude remains much lower that that of copper, so we do not expect this parameter to influence the FCC beam[5].

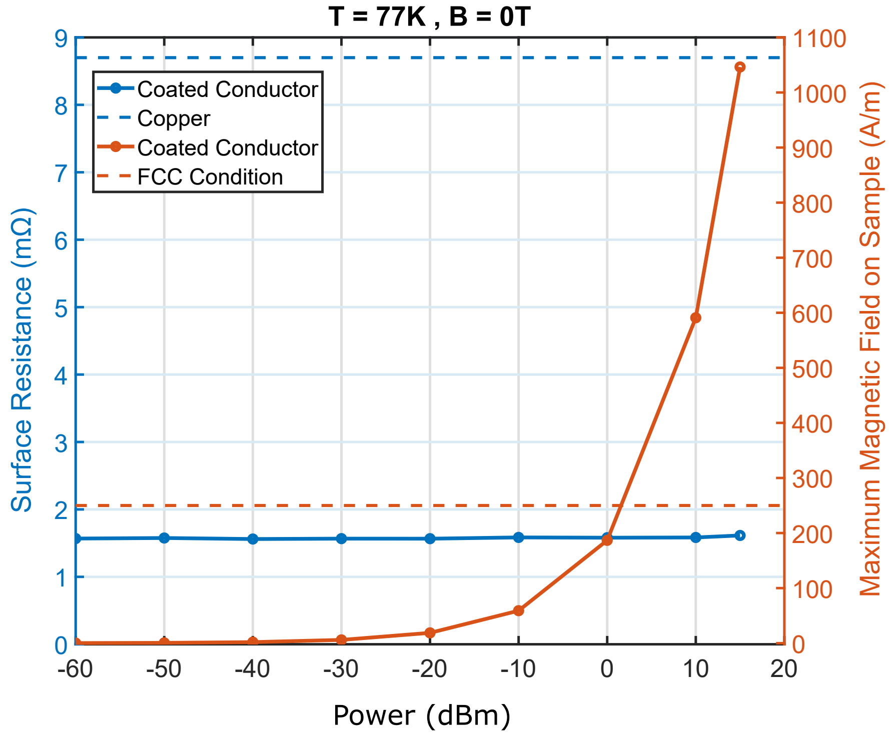

Figure S2. The blue curves represent the surface resistance dependence on the applied power for SuperPower in comparison with copper at 77 K without applied external field. The maximum magnetic field on the sample as a function of the applied power is shown as red solid points. The expected maximum magnetic field on the sample within the FCC-hh is represented by the dashed red line. Adopted from[5].

References

1. Puig, T. *et al.* Coated conductor technology for the beamscreen chamber of future high energy circular colliders. *Supercond. Sci. Technol.* **32**, 094006, DOI: 10.1088/1361-6668/ab2e66 (2019).
2. Lao, M., Bernardi, J., Bauer, M. & Eisterer, M. Critical current anisotropy of GdBCO tapes grown on ISD–MgO buffered substrate. *Supercond. Sci. Technol.* **28**, 124002 (2015).
3. Powell, J. *et al.* Field, temperature, and frequency dependence of the surface impedance of Y-Ba_2-Cu_3-O_7 thin films. *Phys. Rev. B* **57**, 5474 (1998).
4. Wu, D. H., Booth, J. & Anlage, S. M. Frequency and Field Variation of Vortex Dynamics in Y-Ba_2-Cu_3-$O_{7-\delta}$. *Phys. review letters* **75**, 525 (1995).
5. Krkotić, P. *et al.* RF Characterisation of HTS-CC Tapes as Alternative Coating for the FCC-hh Beam Screen (2019). FCC week 2019.
6. Calatroni, S. *et al.* Thallium-based high-temperature superconductors for beam impedance mitigation in the Future Circular Collider. *Supercond. Sci. Technol.* **30**, DOI: 10.1088/1361-6668/aa6bd0 (2017).
